# A combined continuous microflow photochemistry and asymmetric organocatalysis approach for the enantioselective synthesis of tetrahydroquinolines

**DOI:** 10.3762/bjoc.9.284

**Published:** 2013-11-13

**Authors:** Erli Sugiono, Magnus Rueping

**Affiliations:** 1 Institute of Organic Chemistry, RWTH Aachen University, Landoltweg 1, D-52074 Aachen, Germany

**Keywords:** asymmetric transfer hydrogenation, binolphosphate, continuous-flow reactors, flow chemistry, microreactors, organocatalysis, photochemistry

## Abstract

A continuous-flow asymmetric organocatalytic photocyclization–transfer hydrogenation cascade reaction has been developed. The new protocol allows the synthesis of tetrahydroquinolines from readily available 2-aminochalcones using a combination of photochemistry and asymmetric Brønsted acid catalysis. The photocylization and subsequent reduction was performed with catalytic amount of chiral BINOL derived phosphoric acid diester and Hantzsch dihydropyridine as hydrogen source providing the desired products in good yields and with excellent enantioselectivities.

## Introduction

Tetrahydroquinolines [1–4] represent a well-known structural motif found in a large number of biologically active natural products. Optically active tetrahydroquinolines are important building blocks for the pharmaceutical and agrochemical industries. Due to their importance, new and efficient procedures for their synthesis have been developed. Among the synthetic protocols developed for the preparation of optically active tetrahydroquinolines, the asymmetric hydrogenation of substituted quinolines represents the most widely used and efficient method to prepare this class of N-heterocyclic compound [5–17].

In the past years, continuous-flow chemistry has received considerable attention and microstructured continuous-flow devices have emerged as useful devices for different chemical reactions [18–22]. Microreactor technology offers numerous practical advantages such as better reaction yield due to enhanced mixing quality, better control of reaction variables, reduced safety hazards, reduced reagent consumption, enhanced heat and mass transfer due to the high surface-to-volume ratio and rapid experimentation and optimization.

Recently, microreactor devices have been adopted for photochemical applications and microflow photochemistry has emerged as efficient synthesis tool [23–31]. The narrow inner dimensions of microfabricated reactors is advantageous for photochemical synthesis since it allows better light penetration and uniform irradiation through the entire reactor and the complete reaction medium, in comparison with reactions performed in conventional batch systems.

**Scheme 1:**
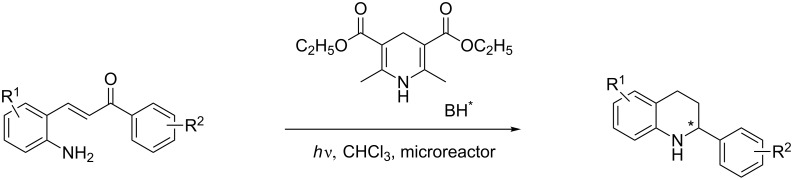
Photocyclization–reduction of 2-aminochalcone.

Here we report the development of continuous-flow photochemical reaction in combination with asymmetric Brønsted acid catalysis for the synthesis of optically active tetrahydroquinolines. Readily available substituted 2-aminochalcones were envisioned to undergo photocyclization to the corresponding quinolines which in the presence of a chiral BINOL-derived phosphoric acid diester and Hantzsch dihydropyridine as hydride donor [32–37] could provide the desired enantioenriched tetrahydroquinolines (Scheme 1) [38].

## Results and Discussion

**Scheme 2:**
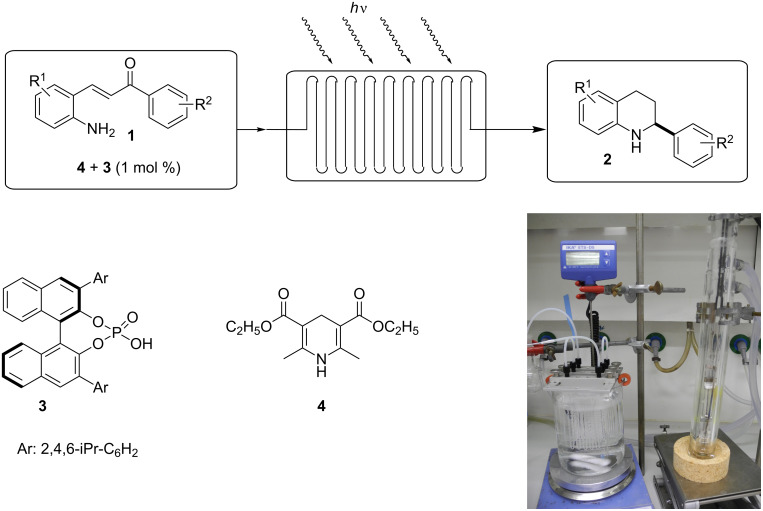
Experimental setup of continuous-flow photocyclization–reduction cascade.

The continuous-flow microreactor system for the experiment was set up according to Scheme 2. The flow device was set up with multiple commercially available glass reactors connected in parallel and placed in a water bath [39]. The light required to perform the reaction is supplied from a high-pressure mercury lamp located outside of the reactor. The lamp consists of a double-jacketed water-cooled pyrex immersion well. The reagents were degassed and introduced into the microreactor using a programmable syringe pump. The product solution was collected in a flask wrapped with aluminium foil to prevent further irradiation.

Our initial investigation of reaction conditions involved the photocyclization–reduction cascade of 2-aminochalcone **1a** in the presence of Hantzsch dihydropyridine **4** as hydrogen source and catalytic amount of chiral Brønsted acid **3**. The effect of temperature, flow rate and concentration on the reaction yield and enantioselectivity are summarized in Table 1. As shown in Table 1, performing the reaction in a pyrex test tube (i.d.: 12 mm; λ > 300 nm) with 1 mol % of Brønsted acid **3** at 40 °C for 60 min afforded the product in 7% isolated yield and 95% enantioselectivity (Table 1, entry 2). Conducting the reaction using the same light source and under the same reaction conditions in a single pass flow reaction showed a noticeable impact on the yield as the product **2a** could be isolated in 59% yield and 93% enantiomeric excess (Table 1, entry 1 vs entry 2). Improvement of the reaction yield shows the superior performance of the microflow reactor since the light penetration through the microchannels was significantly increased. A slight improvement of yield was achieved when the reaction was carried out at 55 °C (Table 1, entry 3).

**Table 1:** Optimization of the Brønsted acid catalyzed transfer hydrogenation of quinolines.^a^

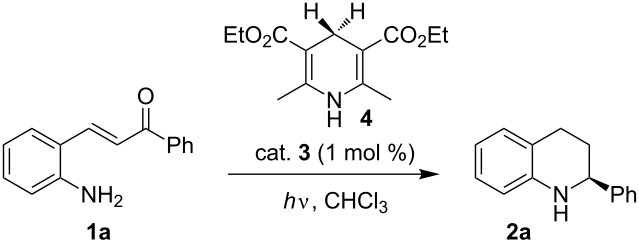						

Entry	Conc. [mol/L]	Temp. [°C]	Time [min]	Flow rate [mL min^−1^]	Yield [%]^b^	ee [%]^c^

1	0.1	40	60	0.1	59	93
2^d^	0.1	40	60	batch	7	95
3	0.1	55	60	0.1	64	96
4	0.05	55	60	0.1	74	94
5	0.05	55	120	0.05	79	88
6	0.03	55	60	0.1	82	94
7^d^	0.03	55	60	batch	29	96
8	0.03	55	120	0.05	88	83

^a^Reaction conditions: **1a**, **4** (2.4 equiv), **3** (1 mol %) in CHCl_3,_ irradiation with a TQ 150 high pressure mercury lamp. ^b^Isolated yields after column chromatography. ^c^Determined by chiral HPLC analysis. ^d^Performed under batch condition.

Noticeable improvement on the chemical yield was observed when the reaction was conducted at a lower concentration providing the product in 74% isolated yield and 94% enantiomeric excess (Table 1, entry 4 vs entry 3). Further decrease of the concentration to 0.03 M gave the best result affording the product in 82% yield (Table 1, entry 6). It is worth mentioning that decreasing the flow rate had only a minimum effect on the yield but resulted in significant loss of enantioselectivity (Table 1, entry 5 vs 4). This result indicates that the residence time plays a crucial role in this photocyclization–reduction cascade. Due to prolonged irradiation of the reaction mixture, an undesired background reaction initiated by photoexcited dihydropyridine occurred leading to the loss of enantioselectivity [40–41].

With the optimized reaction conditions in hand, the substrate scope of this new photocyclization–asymmetric transfer hydrogenation sequence was examined. The results are summarized in Table 2. In general, different 2-aminochalcones bearing substituted aromatic residues on both ketone and enone moieties underwent the desired photocyclization and subsequent asymmetric reduction to afford the corresponding tetrahydroquinolines in good yields and high enantioselectivities.

**Table 2:** Scope of the continuous-flow photocyclization–asymmetric reduction domino sequence.^a^

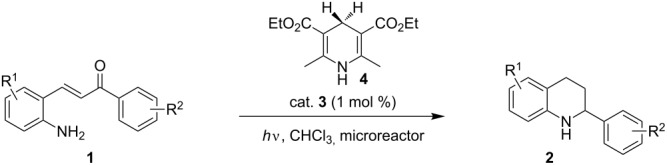				

Entry^a^	Substrate **1**	Product **2**	Yield [%]^b^	ee [%]^c^

1	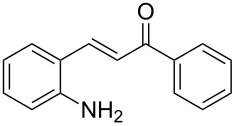 **1a**	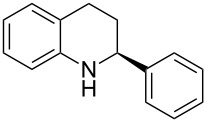 **2a**	82	94
2	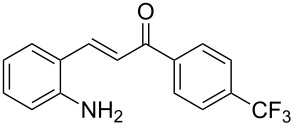 **1b**	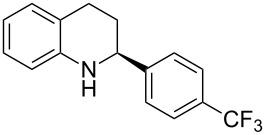 **2b**	88	96
3	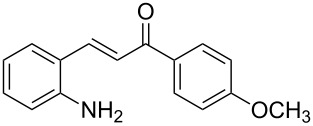 **1c**	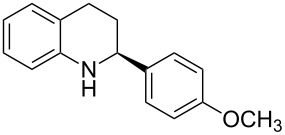 **2c**	73	91
4	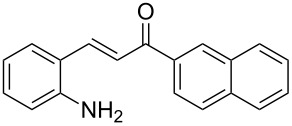 **1d**	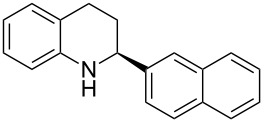 **2d**	71	91
5	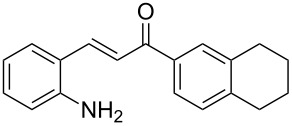 **1e**	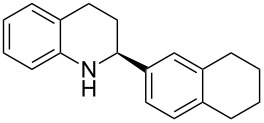 **2e**	63	89
6	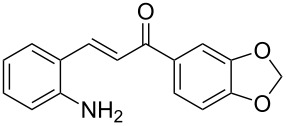 **1f**	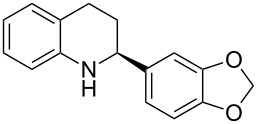 **2f**	73	90
7	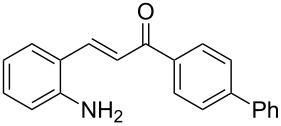 **1g**	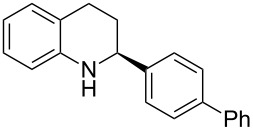 **2g**	75	88
8	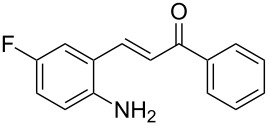 **1h**	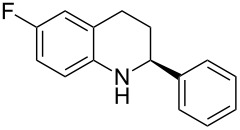 **2h**	64	90
9	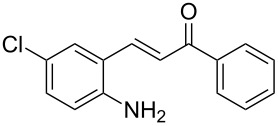 **1i**	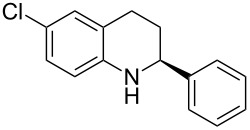 **2i**	57	91

^a^Reaction conditions: **1**, **4** (2.4 equiv), **3** (1 mol %) in CHCl_3_ (0.03 M) at 55 °C, flow rate 0.1 mL/min, residence time = 60 min, irradiation with a TQ 150 high pressure mercury lamp. ^b^Isolated yields after column chromatography. ^c^Determined by chiral HPLC analysis.

## Conclusion

In conclusion, we have demonstrated the great potential of a new continuous-flow microreactor system for the photocyclization–reduction cascade of 2-aminochalcones. Under the continuous-flow condition a variety of substituted 2-aminochalcones underwent the photocyclization and the subsequent transfer hydrogenation to afford a series of differently substituted tetrahydroquinolines in good yields and with excellent enantioselectivities. This efficient protocol for the synthesis of tetrahydroquinoline from readily available 2-aminochalcone provides an attractive alternative to the existing procedures and serves as a basis for further exploration of this new concept.
